# Reduced Aqueous Humor TGF-β2 Levels in Diabetic Cataract: A Comparative Analysis with NF-κB

**DOI:** 10.3390/jcm15124807

**Published:** 2026-06-21

**Authors:** Duygu Tozcu Yilmaz, Mehmet Ali Gul, Mustafa Capraz, Melek Tufek, Nihat Aydin

**Affiliations:** 1 Department of Physiology, Faculty of Medicine, Amasya University, Amasya 05100, Türkiye; 2 Department of Medical Biochemistry, Faculty of Medicine, Amasya University, Amasya 05100, Türkiye; mehmetali.gul@amasya.edu.tr; 3 Department of Internal Diseases, Faculty of Medicine, Amasya University, Amasya 05100, Türkiye; m.capraz@amasya.edu.tr; 4 Department of Ophthalmology, Faculty of Medicine, Amasya University, Amasya 05100, Türkiye; melek.tufek@amasya.edu.tr (M.T.); nihat.aydin@amasya.edu.tr (N.A.)

**Keywords:** diabetic cataract, aqueous humor, TGF-β2, NF-κB, ocular immune privilege

## Abstract

**Background/Objectives:** Type 2 diabetes may impair anterior segment immune regulation. Because transforming growth factor-β2 maintains ocular immune privilege, while nuclear factor-κB is linked to inflammatory activation, we compared their aqueous humor levels in cataract patients with and without diabetes. **Methods:** In this prospective cross-sectional study, aqueous humor samples were collected from 90 patients (30 diabetic, 60 non-diabetic) via anterior chamber needle aspiration at the commencement of routine phacoemulsification, prior to viscoelastic injection, without additional intervention. Transforming growth factor-β2 and nuclear factor-κB levels were then measured using enzyme-linked immunosorbent assay (ELISA). Between-group comparisons and ROC curve analyses were performed to evaluate differences in biomarker levels and their discriminative ability in distinguishing diabetic status. Covariate-adjusted analysis (ANCOVA) was additionally performed. **Results:** Transforming growth factor-β2 levels were significantly lower in the diabetic group (*p* < 0.001), while nuclear factor-κB levels showed no significant difference (*p* = 0.285). The between-group difference in transforming growth factor-β2 remained significant after adjustment for cataract grade and hypertension duration (F(1,86) = 17.901, *p* < 0.001, partial η^2^ = 0.172; Cohen’s d = 0.94). Transforming growth factor-β2 demonstrated high specificity (100%) but limited sensitivity (45%) for identifying diabetic status at a cut-off of <449.25 ng/L; however, given the small sample size and exploratory nature of the study, this specificity value should be interpreted with caution and requires validation in larger cohorts. **Conclusions:** Lower aqueous humor TGF-β2 levels in diabetic cataract patients, independent of cataract severity and hypertension duration, suggest that TGF-β2 suppression may represent an earlier molecular event in anterior segment immune dysregulation preceding overt inflammatory activation. While TGF-β2 shows exploratory biomarker potential, validation in larger, prospective, mechanistic studies is required before clinical application.

## 1. Introduction

Despite the well-established association between type 2 diabetes mellitus and accelerated cataract development [1,2], the molecular mechanisms underlying this relationship remain incompletely understood. While structural lens changes in diabetic patients have been extensively characterized, the impact of diabetes on the immune microenvironment of the anterior segment—particularly the balance between immunosuppressive and pro-inflammatory mediators in the aqueous humor—has received comparatively little attention [3,4]. Transforming growth factor-β2 (TGF-β2), the predominant isoform in aqueous humor, is a critical mediator of ocular immune privilege [5,6], whereas nuclear factor-κB (NF-κB) represents a key driver of inflammatory activation [7,8]. Whether diabetes disrupts the homeostatic balance between these two pathways in the anterior segment prior to clinically manifest inflammation remains unknown. To address this question, we conducted a prospective cross-sectional observational study comparing TGF-β2 and NF-κB levels in the aqueous humor of diabetic and non-diabetic cataract patients.

Aqueous humor is not merely a refractive medium; it is a biochemically active fluid that may reflect the metabolic and immunological state of the anterior segment [9,10]. Its composition—comprising growth factors, cytokines, and regulatory proteins—is altered in systemic metabolic diseases such as diabetes, yet the specific consequences for anterior segment immune regulation are poorly defined [3,11]. The anterior segment is considered an immune-privileged region, and this delicate balance is maintained by immunomodulatory factors within the aqueous humor [12]. Although metabolic and oxidative stress associated with diabetes may affect this microenvironment [13,14], most existing studies have focused on posterior segment disease and advanced diabetic ocular complications, leaving early anterior segment immune dysregulation in diabetic cataract insufficiently characterized [4,15]. Furthermore, inconsistencies in the existing literature may reflect heterogeneity in patient cohorts, including diabetic retinopathy versus cataract-only populations, disease stage, and whether total or active TGF-β2 was assessed.

TGF-β2 is a critical cytokine that prevents tissue damage by providing an immunosuppressive microenvironment in ocular tissues [5,6]. It exerts bidirectional regulatory effects on cell growth and differentiation depending on tissue source, cell type, and microenvironment [16,17], and TGF-β2 signalling in lens tissue has been implicated in both physiological processes and pathological conditions including cataractogenesis [18]. In cataract-only cohorts, aqueous humor TGF-β2 levels have been reported to increase in proportion to cataract severity [18,19]. In contrast, studies focusing on diabetic retinopathy cohorts have reported elevated TGF-β2 concentrations, likely reflecting fibrotic and neovascular processes predominant in advanced posterior segment disease [20,21]. Importantly, a limited number of studies have reported higher TGF-β1 and TGF-β2 concentrations in diabetic cataract patients compared to age-related cataract patients [20], though findings remain inconsistent across cohorts.

An important methodological consideration across TGF-β2 studies is whether total or biologically active TGF-β2 was measured. TGF-β2 exists predominantly in a latent form in aqueous humor, and its activation depends on the local microenvironment—including integrin-mediated mechanisms and the degree of fibrotic signalling [16]. Studies measuring only total TGF-β2 may therefore not capture functional differences in TGF-β2 activity, which may partly explain inconsistencies in the literature. Conversely, NF-κB is a pivotal regulator of the inflammatory response. The NF-κB signaling pathway plays a critical role in the pathogenesis of diabetes-related vascular complications, triggering cellular stress responses by increasing proinflammatory cytokine expression [7,8]. It is acknowledged that diabetes has the capacity to modify the immune-privileged structure of the eye by disrupting the blood-aqueous barrier and activating inflammatory signalling pathways [22].

While the structural effects of diabetes on the lens have been comprehensively explored, data concerning the impact of diabetes on the balance between immunomodulatory and inflammatory pathways in the anterior segment remains limited. In the context of diabetic cataract patients, the molecular mechanisms that alter ocular immune privilege prior to the development of a clinically apparent inflammatory response remain to be elucidated. We hypothesised that type 2 diabetes is associated with reduced aqueous humor TGF-β2 levels and altered NF-κB-related inflammatory signalling, reflecting disruption of anterior segment immune homeostasis before clinically overt inflammation becomes apparent. The present observational study addresses this question by comparing TGF-β2 and NF-κB levels in the aqueous humor of diabetic and non-diabetic cataract patients, with the aim of characterizing diabetes-associated alterations in anterior segment immune homeostasis.

## 2. Materials and Methods

### 2.1. Study Design and Populations

This prospective cross-sectional study was conducted at the Department of Internal Medicine and Ophthalmology of Amasya University Sabuncuoğlu Şerefeddin Training and Research Hospital between October 2022 and August 2023. The study population comprised 90 participants, who were divided into two groups: 30 diabetic cataract patients and 60 non-diabetic cataract patients. The sample size was not determined by an a priori power calculation because of the exploratory nature of the study and the limited availability of aqueous humor samples. All eligible patients who met the inclusion criteria and provided consent during the study period were consecutively included. Based on the observed effect size for TGF-β2 (Cohen’s d = 0.94), a post hoc power calculation indicated 98.6% power for detecting the observed between-group difference (α = 0.05, two-tailed). The diagnosis of type 2 diabetes was made in accordance with the American Diabetes Association (ADA) criteria. Cataract grade was assessed using the Lens Opacities Classification System II (LOCS II) by two independent ophthalmologists; in cases of discrepancy, consensus was reached by joint re-evaluation. Detailed diabetes-related parameters, including HbA1c levels, diabetic retinopathy status, and antidiabetic medication use, were not systematically available for all participants and were therefore not included in the analysis. Exclusion criteria encompassed the following: age < 18, renal/hepatic failure, rheumatologic or inflammatory diseases, thyroid dysfunction, heart failure, chronic infection, pancreatic disorders, use of drugs affecting insulin secretion/sensitivity, pregnancy or lactation, and unwillingness to participate.

### 2.2. Sample Collection and Storage

Aqueous humor samples were obtained at the commencement of standard phacoemulsification cataract surgery. Following the standard preparation of the surgical field, lateral corneal incisions were created using a 15-degree blade. Prior to the administration of any viscoelastic substance into the anterior chamber, aqueous humor was aspirated through a 30-gauge anterior chamber needle. Approximately 100 μL of aqueous humor was carefully collected from each eye and expeditiously transferred into sterile Eppendorf tubes. During sample collection, care was taken to avoid contact with the conjunctiva, cornea, iris, lens capsule, or lens tissue to prevent contamination. All collected samples were immediately frozen without delay and stored at −80 °C until biochemical analysis.

### 2.3. Biochemical Analysis

The concentrations of aqueous humor TGF-β2 and NF-κB were determined using ELISA kits (Bioassay Technology Laboratory, Shanghai, China; Cat. No. E0067Hu and E0690Hu, respectively). Absorbance readings were performed with a Chromate 4300 ELISA reader (Awareness Technology, Inc., Martin Hwy., Palm City, USA) at a wavelength of 450 nm. The results were expressed in ng/L for TGF-β2 and ng/mL for NF-κB, in accordance with the respective manufacturer’s kit specifications. Although NF-κB is primarily an intracellular transcription factor, its measurement in aqueous humor was performed as an exploratory approach to assess extracellular inflammatory signaling within the anterior segment microenvironment. Aqueous humor sampling during cataract surgery provides a clinically accessible matrix for evaluating local ocular inflammatory activity. Therefore, NF-κB values in this study should be interpreted as exploratory extracellular protein measurements rather than as a direct measure of intracellular NF-κB activation. The intra-assay coefficients of variation (CV) for TGF-β2 and NF-κB kits were below 7.5% and 6.8%, respectively, indicating acceptable precision. Standard curves for both assays demonstrated linearity with R^2^ = 0.99. Both assays measure total protein levels; in the case of TGF-β2, which exists predominantly in latent form in aqueous humor, measured concentrations reflect total rather than biologically active TGF-β2.

### 2.4. Statistical Analysis

The data analysis was conducted utilising SPSS version 22 (SPSS Inc., Chicago, IL, USA). The statistical significance was set at *p* < 0.05 (two-tailed). The assessment of normality was conducted through the utilisation of Kolmogorov–Smirnov and Shapiro–Wilk tests. The Independent-Samples *t* test was employed for the purpose of conducting comparisons between groups for variables that were normally distributed and continuous, with the results of this analysis being presented as ‘mean ± standard deviation (SD)’. In instances where continuous variables did not demonstrate a normal distribution, the Mann–Whitney U-test was employed for the purpose of intergroup comparisons, with the results being presented as ‘median and interquartile range (IQR)’. The qualitative data were then subjected to a Chi-square test of independence, and the results were presented as a percentage. To control for potential confounding effects of cataract grade and hypertension duration on TGF-β2 levels, a one-way ANCOVA was performed with group (diabetic vs. non-diabetic) as the fixed factor and cataract grade and hypertension duration as covariates. Prior to ANCOVA, the homogeneity of regression slopes assumption was verified by testing Group × covariate interaction terms (Group × cataract grade: F(1,84) = 0.999, *p* = 0.321; Group × hypertension duration: F(1,84) = 1.134, *p* = 0.290), and residual normality was confirmed using the Shapiro–Wilk test (W = 0.984, *p* = 0.344), indicating that ANCOVA assumptions were adequately met. Effect sizes were calculated using Cohen’s d for independent group comparisons, with values of 0.2, 0.5, and 0.8 interpreted as small, medium, and large effects, respectively. Receiver operating characteristic (ROC) curve analysis was performed to explore the discriminative ability of aqueous humor TGF-β2 levels between diabetic and non-diabetic cataract patients. The optimal cut-off value was determined using the Youden index (sensitivity + specificity − 1).

## 3. Results

**Figure 1:**
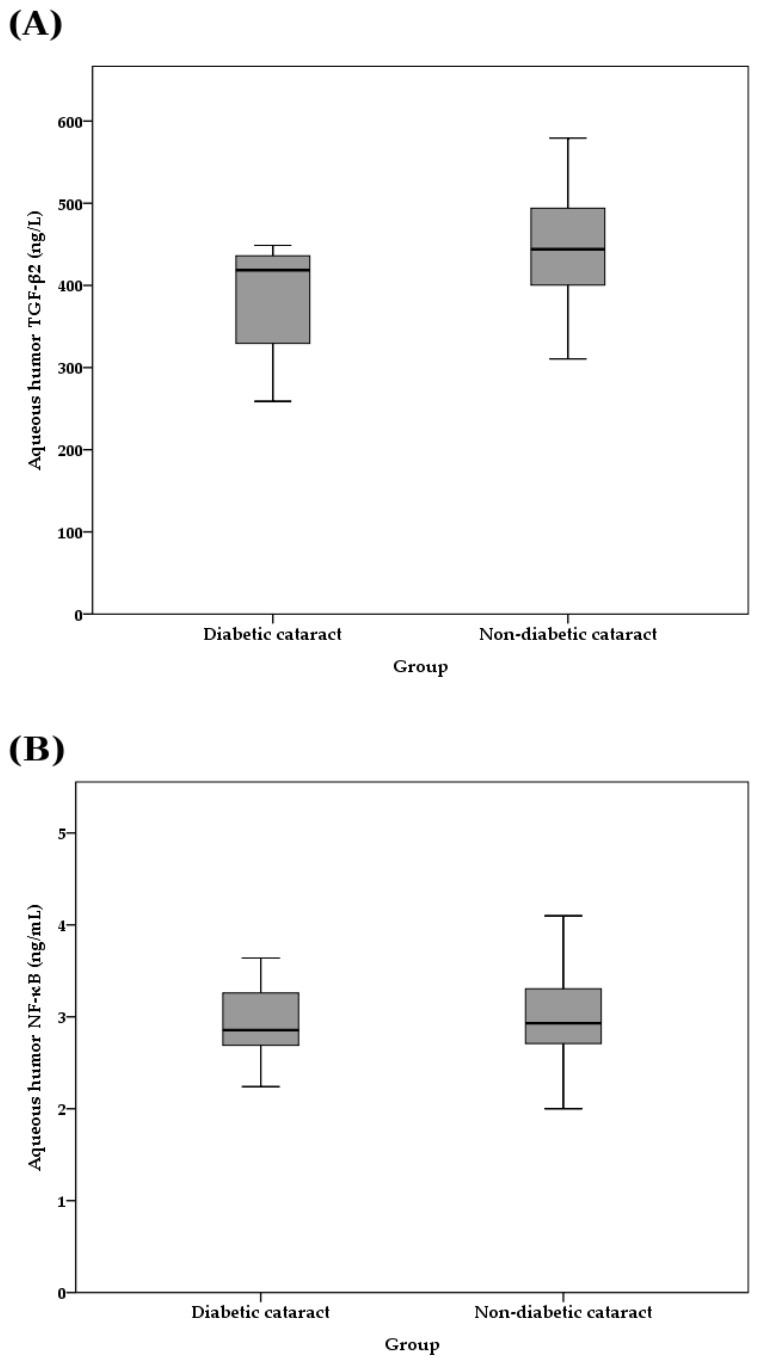
Aqueous humor biomarker levels in diabetic and non-diabetic cataract patients. (**A**) TGF-β2 levels were significantly lower in diabetic cataract patients compared with non-diabetic cataract patients (*p* < 0.001). (**B**) NF-κB levels did not differ significantly between groups (*p* = 0.285). Boxes indicate the interquartile range with median lines.

Levels of TGF-β2 in the diabetic cataract group were significantly lower than in the non-diabetic cataract group (376.56 ± 68.78 ng/L vs. 443.53 ± 72.53 ng/L; mean difference: −66.97 ng/L, 95% CI: −98.66 to −35.28; *p* < 0.001; Cohen’s d = 0.94). No statistically significant difference was identified between the groups with respect to NF-κB levels (2.93 ± 0.38 ng/mL vs. 3.03 ± 0.47 ng/mL; mean difference: −0.10 ng/mL, 95% CI: −0.301 to 0.090; *p* = 0.285; Cohen’s d = 0.23). These between-group differences are illustrated in Figure 1.

A comparative analysis of clinical parameters revealed that the degree of cataract was significantly higher in the non-diabetic cataract group compared to the diabetic cataract group (2.97 ± 0.92 vs. 2.2 ± 0.81, *p* < 0.001). Furthermore, the duration of hypertension was found to be longer in the diabetic cataract group (11.57 ± 5.59 years vs. 6.22 ± 6.34 years, *p* < 0.001). No significant between-group differences were observed in age (69.47 ± 7.64 vs. 70.53 ± 9.82 years, *p* = 0.604), visual acuity (0.2 [0.9] vs. 0.2 [0.79], *p* = 0.385), or axial length (23.01 ± 0.74 vs. 23.28 ± 0.86 mm, *p* = 0.145) (*p* > 0.05) (Table 1). These baseline differences in cataract grade and hypertension duration were considered as potential confounders and were addressed in the covariate-adjusted analysis and in the limitations section.

To examine whether the between-group difference in TGF-β2 levels was independent of cataract severity and hypertension duration, a one-way ANCOVA was conducted with group as the fixed factor and cataract grade and hypertension duration as covariates. Cataract grade exerted a significant effect on TGF-β2 levels (F(1,86) = 4.328, *p* = 0.040, partial η^2^ = 0.048), whereas hypertension duration did not (F(1,86) = 0.385, *p* = 0.537, partial η^2^ = 0.004). Critically, the group effect remained statistically significant after adjustment for both covariates (F(1,86) = 17.901, *p* < 0.001, partial η^2^ = 0.172), indicating that lower TGF-β2 levels in the diabetic cataract group persisted after adjustment for cataract grade and hypertension duration (Table 2). The magnitude of the difference in TGF-β2 levels between groups was large (Cohen’s d = 0.94), whereas the difference in NF-κB levels represented a small effect size (Cohen’s d = 0.23), consistent with the absence of statistical significance for the latter.

**Figure 2:**
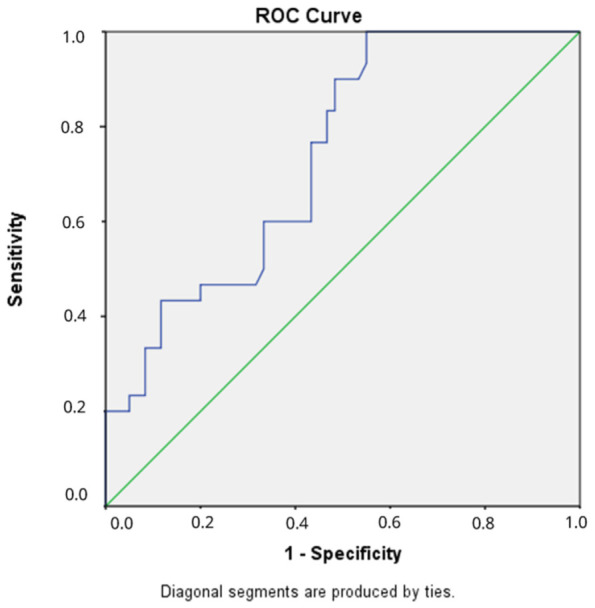
Receiver operating characteristic (ROC) curve showing the performance of aqueous humor TGF-β2 levels in distinguishing diabetic and non-diabetic cataract patients. The blue line represents the ROC curve for aqueous humor TGF-β2, whereas the green diagonal line represents the reference line for a non-discriminatory test (area under the curve = 0.50).

A receiver operating characteristic (ROC) curve analysis was performed to evaluate the discriminative ability of aqueous humor TGF-β2 levels to distinguish between diabetic and non-diabetic cataract patients. The area under the curve (AUC) for TGF-β2 was determined to be 0.737 (95% CI: 0.635–0.840; *p* < 0.001). At the cutoff point of <449.25 ng/L, determined using the Youden index, the sensitivity of TGF-β2 was 45%, and its specificity was 100% (Figure 2). However, the 100% specificity observed at this cut-off should be interpreted with caution, as such extreme values are prone to overestimation in relatively small sample sizes and require external validation in independent cohorts.

## 4. Discussion

While it is recognised that individuals with diabetes are predisposed to developing cataracts and that the progression of the disease is more rapid in comparison to non-diabetic individuals, the clinical manifestations of diabetic and non-diabetic cataract patients remain largely analogous; the molecular and biochemical mechanisms underpinning this disparity have yet to be fully elucidated [20]. The majority of extant studies on the pathogenesis of diabetic cataract have focused on posterior segment involvement and advanced diabetic ocular complications, while the role of immune and inflammatory regulators located in the anterior segment of the eye has been investigated in a relatively limited number of studies [3,4,15,23]. This necessitates the investigation of biochemical changes caused by diabetes in the microenvironment of the anterior segment of the eye in the pathogenesis of cataracts [19,21,24]. In particular, further research is required to elucidate the changes in cytokines and transcription factors within the aqueous humor microenvironment in the presence of diabetes, and to establish the relationship between these changes and cataract development [20,25,26].

In this context, the present study compared the levels of TGF-β2 and NF-κB, two important components of the immune regulatory and inflammatory response, in the aqueous humor of diabetic and non-diabetic cataract patients. The findings of the present study demonstrated that levels of TGF-β2 were significantly lower in diabetic cataract patients when compared to non-diabetic cataract patients, whilst NF-κB levels did not differ between the two groups. TGF-β2 is a cytokine that is predominantly found in the aqueous humor in a latent (inactive) form and at high concentrations [27]. This molecule, which is widely expressed in eye tissues, is defined as a pleiotropic growth factor that regulates the proliferation, migration, and differentiation of lens epithelial cells and plays a role in morphological plasticity and epithelial–mesenchymal transition (EMT) processes [28,29]. Furthermore, TGF-β2 plays a pivotal role in the maintenance of immune tolerance against inflammatory stimuli and the preservation of ocular tissue homeostasis [6]. It has been reported that TGF-β2 can exhibit a dual function in lens epithelial cells depending on the situation [16]. Within the same cell population, TGF-β2 has been observed to trigger the transformation of some cells into the myofibroblast phenotype, while inducing the differentiation of other cells into lens fiber cells. These differentiation responses have been associated with the activation of the p38/MAPK and mTOR signaling pathways, respectively [28]. A review of the extant literature reveals a consensus that levels of TGF-β2 in the aqueous humor of cataract patients are increased in comparison to those observed in healthy individuals [18,19]. Furthermore, a limited number of studies have reported that TGF-β1 and TGF-β2 concentrations are higher in diabetic cataract patients compared to age-related cataract patients [20].

In the present study, the findings that TGF-β2 levels in the aqueous humor of diabetic cataract patients were lower compared to non-diabetic cataract patients partially differ from the literature. In interpreting this difference, it is important to note that the degree of cataract in the non-diabetic cataract group was significantly higher than in the diabetic group. Indeed, studies in the literature demonstrate that aqueous humor TGF-β2 levels increase in proportion to the severity of cataract [18].

This finding suggests that the advanced stage of cataracts observed in the non-diabetic cataract group may have contributed to the elevated TGF-β2 levels detected in the aqueous humor. Conversely, the lower degree of cataract in the diabetic cataract group, coupled with the suppression of TGF-β2 levels, suggests that the diabetic microenvironment may modulate TGF-β signaling independently of cataract severity. The biological activity of TGF-β2 is contingent not only on its absolute concentration, but also on its capacity to convert to the active form within the microenvironment [16]. Experimental studies on lens tissue have demonstrated that latent TGF-β is activated via αvβ3 integrin in hard and fibrotic wound microenvironments, leading to a pronounced fibrotic response. Conversely, TGF-β activation remains limited in physiological and non-fibrotic environments where the basement membrane is preserved [16]. In this context, the low TGF-β2 levels detected in the aqueous humor of diabetic cataract patients suggest that TGF-β activation may be suppressed in a microenvironment of the anterior segment that is non-fibrotic but dominated by chronic metabolic stress. Furthermore, the sustained hypertension observed in the diabetic group may have resulted in elevated chronic vascular and metabolic stress in the anterior segment, potentially leading to the progressive suppression of TGF-β signalling over time. It has previously been reported that the coexistence of hypertension and diabetes can modulate TGF-β/SMAD pathways through oxidative stress and endothelial dysfunction, and alter tissue-specific TGF-β responses [8,21]. When these mechanisms are considered collectively, it is hypothesised that the low TGF-β2 levels observed in diabetic cataract patients may reflect the combined effect of both cataract severity and systemic microenvironmental factors associated with diabetes. Importantly, covariate-adjusted analysis (ANCOVA) confirmed that the group difference in TGF-β2 levels persisted independently of both cataract grade and hypertension duration (F(1,86) = 17.901, *p* < 0.001, partial η^2^ = 0.172), demonstrating that these between-group differences do not account for the observed TGF-β2 suppression in diabetic patients.

NF-κB is a key pro-inflammatory transcription factor that regulates the expression of cytokines such as IL-1β, TNF-α, and IL-6. It has been established that the activation of this pathway exerts an influence on the development of cataracts [30]. In the context of cataract pathogenesis, it has been documented that endoplasmic reticulum (ER) stress, which arises from impaired cellular protein homeostasis, and the unfolded protein response (UPR), activated in response to this stress, modulate inflammatory and cellular stress responses in close interaction with NF-κB [31]. Recent experimental animal studies have demonstrated that the nuclear factor kappa-B (NF-κB) transcription factor plays a central role in cataract pathogenesis [32,33]. Human studies on cataracts and NF-κB are quite limited in the literature. NF-κB levels are significantly elevated in diabetic patients, leading to sustained pro-inflammatory gene expression in lens epithelial cells under conditions of chronic hyperglycemia [30]. The present study revealed no significant difference in NF-κB levels between the groups. This finding may reflect the stage-dependent nature of NF-κB activation in diabetic ocular disease [30]. In early or moderate diabetic cataract, chronic hyperglycemia may not yet have generated sufficient oxidative stress to trigger sustained NF-κB activation in the anterior segment. Additionally, the blood-aqueous barrier, although disrupted in advanced diabetes, may remain partially intact at this disease stage, partially protecting the aqueous humor compartment from systemic inflammatory signals [22]. It is also plausible that residual TGF-β2 activity may suppress NF-κB activation despite ongoing metabolic stress [6]. Taken together, these considerations suggest that TGF-β2 suppression may precede overt NF-κB-mediated inflammatory activation as an earlier molecular event in diabetic anterior segment dysregulation.

ROC curve analysis revealed an AUC of 0.737 (95% CI: 0.635–0.840), indicating moderate discriminative ability of aqueous humor TGF-β2 in distinguishing diabetic from non-diabetic cataract patients. While the high specificity suggests that TGF-β2 levels below the identified cut-off of 449.25 ng/L may reliably indicate diabetic status, the modest sensitivity of 45% limits its standalone diagnostic utility. Furthermore, the 100% specificity observed at this cut-off should be interpreted with caution, as such extreme values are prone to overestimation in relatively small sample sizes and require external validation in independent cohorts. These findings should be regarded as exploratory, and prospective validation in larger, independent cohorts is necessary before any clinical application can be considered.

The present study should be interpreted within the context of several limitations. First, this was a single-center study with a relatively limited sample size. Although consecutive recruitment was used to reduce selection bias, the single-center design may limit the generalizability of the findings to other clinical settings and patient populations. Therefore, the present findings should be considered exploratory and hypothesis-generating rather than definitive. Validation in larger, multicenter, and longitudinal cohorts with more heterogeneous patient populations is warranted to validate and further clarify these findings. Samples were taken at a single time point during surgery; therefore, temporal changes in biomarker levels could not be monitored. Longitudinal assessment of aqueous humor TGF-β2 and NF-κB levels across different stages of diabetic disease progression would be necessary to determine whether the observed TGF-β2 suppression reflects a persistent alteration of the diabetic anterior segment microenvironment or a dynamic response to metabolic stress. Although covariate-adjusted analysis indicated that the group difference in TGF-β2 levels persisted independently of cataract grade and hypertension duration, residual confounding related to disease severity cannot be entirely excluded. Additionally, detailed diabetes-related parameters including HbA1c levels, diabetic retinopathy status, glycemic control indices, and antidiabetic medication use were not systematically collected. These variables may influence aqueous humor TGF-β2 levels and represent potential confounders that could not be adjusted for in the current analysis. Future studies should incorporate comprehensive diabetes characterization to allow a more precise assessment of the relationship between glycemic burden and anterior segment immune dysregulation. Only protein levels were evaluated, which limits mechanistic interpretations. Furthermore, as the ELISA kit used in this study measures total TGF-β2 rather than the biologically active form, the functional significance of the observed concentration differences cannot be directly inferred. Studies incorporating active TGF-β2 measurement or downstream signaling markers would provide greater mechanistic insight. Multiple clinical and biochemical parameters were compared between groups; however, formal correction for multiple comparisons was not applied, as TGF-β2 was the primary biomarker of interest. Furthermore, the absence of a healthy control group without cataract limits the ability to establish reference ranges for aqueous humor TGF-β2 and NF-κB levels and to determine whether the observed values reflect diabetes-specific changes or cataract-related alterations more broadly. Therefore, larger, mechanistic studies are required. The NF-κB ELISA kit (E0690Hu) was validated by the manufacturer for serum, plasma, urine, ascites, cerebrospinal fluid, and tissue samples. Although aqueous humor is a biological fluid sharing compositional similarities with plasma ultrafiltrate, it was not among the matrices explicitly validated for this kit. This represents a methodological limitation, and NF-κB measurements in aqueous humor should be interpreted accordingly. Moreover, NF-κB is primarily an intracellular transcription factor, and its detection in aqueous humor by ELISA reflects secreted or released protein rather than direct cellular activation status. As such, aqueous humor NF-κB levels may not fully capture the magnitude of inflammatory signaling occurring within anterior segment tissues. Future studies incorporating cellular or tissue-based NF-κB activation assays would provide more direct evidence of inflammatory pathway engagement.

The ROC analysis yielded only moderate discriminative ability (AUC = 0.737), and the observed 100% specificity at the identified cut-off is likely subject to overestimation given the relatively small sample size. These biomarker findings should therefore be regarded as hypothesis-generating, and the diagnostic utility of aqueous humor TGF-β2 requires prospective evaluation in larger, independent cohorts before any clinical conclusions can be drawn.

## 5. Conclusions

This study demonstrates that type 2 diabetes is associated with significantly lower aqueous humor TGF-β2 levels in cataract patients after adjustment for cataract severity and hypertension duration. The absence of a corresponding change in NF-κB levels suggests that TGF-β2 suppression may represent an earlier molecular event in diabetic anterior segment dysregulation, preceding overt inflammatory activation. These findings indicate that diabetes alters the immune homeostasis of the anterior segment in a manner not fully reflected by conventional inflammatory markers. While TGF-β2 shows exploratory biomarker potential, its moderate discriminative ability and the limitations of the current study design necessitate validation in larger, prospective, mechanistic studies before any clinical conclusions can be drawn.

## Figures and Tables

**Table 1:** Demographic, clinical, and biochemical characteristics of diabetic and non-diabetic cataract patients.

	Diabetic Cataract Group (*n* = 30)	Non-Diabetic Cataract Group (*n* = 60)	*p* Values
Age (year)	69.47 ± 7.64	70.53 ± 9.82	0.604 a
Gender			0.527 b
Male	12 (40%)	36 (60%)	
Female	18 (60%)	24 (40%)	
Diabetes mellitus (year)	11.5 ± 4.56	-	
Hypertension (year)	11.57 ± 5.59	6.22 ± 6.34	<0.001 a
Visual Acuity	0.2 (0.9)	0.2 (0.79)	0.385 c
Cataract grade	2.2 ± 0.81	2.97 ± 0.92	<0.001 a
Axial Length	23.01 ± 0.74	23.28 ± 0.86	0.145 a
NF-κB (ng/mL)	2.93 ± 0.38	3.03 ± 0.47	0.285 a
TGF-β2 (ng/L)	376.56 ± 68.78	443.53 ± 72.53	<0.001 a

a Independent Samples *t*-test. Data are presented as mean ± standard deviation (SD). b Chi-square test. Data are presented as n and percentage (%). c Mann–Whitney U test. Data are presented as median and interquartile range (IQR).

**Table 2:** ANCOVA results for aqueous humor TGF-β2 levels with cataract grade and hypertension duration as covariates.

Source	Sum of Squares	df	Mean Square	F	*p*	Partial η^2^
HT duration	1903.770	1	1903.770	0.385	0.537	0.004
Cataract grade	21,395.419	1	21,395.419	4.328	0.040	0.048
Group	88,487.543	1	88,487.543	17.901	**<0.001**	0.172
Error	425,101.232	86	4943.038			

Bold values indicate statistically significant results (*p* < 0.05).

## Data Availability

The data analysed in this study are not available in a public database but are available from the corresponding author upon reasonable request.

## Abbreviations

The following abbreviations are used in this manuscript:

TGF-β2	Transforming growth factor-beta 2
NF-κB	Nuclear factor kappa B
ADA	American Diabetes Association
ROC	Receiver operating characteristic
AUC	Area under the curve
